# When the Counterpart Chooses the Opposite: The First Mover’s Anticipation and Evaluation of the Final Feedback in Gambles

**DOI:** 10.3389/fnins.2022.948579

**Published:** 2022-07-22

**Authors:** Jiehui Zheng, Lei Wang, Liang Meng

**Affiliations:** ^1^Alibaba Business School, Hangzhou Normal University, Hangzhou, China; ^2^Neuromanagement Lab, Zhejiang University, Hangzhou, China; ^3^School of Management, Zhejiang University, Hangzhou, China; ^4^School of Business and Management, Shanghai International Studies University, Shanghai, China; ^5^Institute of Organizational Behavior and Organizational Neuroscience, Shanghai International Studies University, Shanghai, China

**Keywords:** social information, choice inconsistency, anticipation, outcome evaluation, event-related potentials (ERP), feedback-related negativity (FRN), stimulus-preceding negativity (SPN)

## Abstract

This research examines the effect of response (in)consistency on the first mover’s anticipation and evaluation of the performance feedback in gambles. In a two-player gambling task, the participant played as the first mover while the confederate served as the second mover, who made their gambles in sequence. A more pronounced feedback-related negativity (FRN) was observed when the first mover noticed that the second mover chose a different option from him/her. An enlarged stimulus-preceding negativity (SPN) was observed when the first mover was anticipating the final feedback in this condition. Interestingly, consistent responses gave rise to a more pronounced FRN difference wave (d-FRN) during the feedback stage. Taken together, these results suggest that response discrepancy would modulate the first mover’s anticipation and evaluation of the final feedback in gambles.

## Introduction

During our social interactions with others in daily lives, we receive varied social information, such as others’ viewpoints, behaviors and so on. Social information is ubiquitous, which heavily influences one’s thinking and actions. Imagine you are visiting a popular cake store, which is promoting two kinds of blind boxes. Given the high uncertainty, it is difficult to figure out which box contains the more delicious cake. You decided to buy one of the two blind boxes and made your choice by yourself, after which you happened to learn about the choice of another customer. How would you feel if you found that his or her decision was different from yours? Would this social information of choice inconsistency influence your subsequent cognitive reactions to the decision outcome (i.e., whether your cake was yummy and to your taste)? These questions are attracting researchers’ attention but have not been fully resolved in existing literatures.

On the one hand, some previous research has examined the influence of social information on one’s behaviors and the neural mechanisms underlying one’s evaluation of such social information. It was found that when individuals find out that they provide an inconsistent choice with others, a negative signal in the brain would be generated, which would guide them to perform subsequent behaviors in a certain manner, such as showing conformity (Klucharev et al., 2009; Campbell-Meiklejohn et al., 2010; Schnuerch and Gibbons, 2014). For example, Klucharev et al. (2009) conducted a facial attractiveness judgment task, and found that when individuals find their own opinion to be different from the group’s, they would change their own rating in the direction of the group’s rating. Thus, consistent social information is generally regarded as a reward signal (Campbell-Meiklejohn et al., 2010; Zaki et al., 2011), while social information on inconsistency with others may bring about emotional and cognitive conflicts (Klucharev et al., 2011; Yu and Sun, 2013). This conflict would be processed as a negative feedback and then reflect in the magnitude of the feedback-related negativity (FRN) (Wang et al., 2019; Zheng et al., 2021). In early studies the valence effect on FRN was demonstrated mainly in studies adopting monetary rewards tasks (Gehring and Willoughby, 2002; Yeung and Sanfey, 2004; Liao et al., 2011; San Martín, 2012), with the loss condition eliciting a larger FRN than the gain condition (Yu et al., 2007; Ma et al., 2011; Wang Y. et al., 2016; Qi et al., 2018). In recent years, more and more studies found FRN to be sensitive to the inconsistency in social information. For example, in a recent study, the researchers asked two anonymous same-sex players to work on a knowledge quiz task, and found that inconsistent answers with the partner make the participants feel more uncertain about their own responses, resulting in a larger FRN (Wang et al., 2018). Similarly, Zheng et al. (2021) modified the task of Klucharev et al. (2009) by introducing the crowdfunding context and found that deviation of the individual rating from the group rating evokes a significantly more negative FRN. Given that the above findings are illuminating, most existing studies examined the (in)consistency effect by comparing one’s behavior or opinion with the whole group’s. However, whether consistency between paired individuals would produce a similar social influence needs further examination.

On the other hand, some researchers resorted to the FRN to examine whether the consistency in choices would affect the evaluative processing of decision outcomes (Yu et al., 2007; Kimura and Katayama, 2013; Fu et al., 2017; Kimura et al., 2018). For example, Kimura et al. (2018) found that consistent choices with the group in a cooperative context elicit a smaller d-FRN (FRN _loss_ minus FRN _gain_) than inconsistent choices. Since all participants’ choices contribute to and determine each one’s outcome in a cooperative context, the researchers attributed this result to the diffusion of responsibility for the outcome, which makes the outcome less relevant to each participant and decreases the motivational value of the outcome. Another study reported similar results that reduced d-FRN is elicited during outcome evaluation when the participants make the same decision as others (Yu and Sun, 2013). The authors speculated that individuals would experience less negative emotion toward the loss outcome when they are consistent with the crowd. However, a contrary finding was reported by Fu et al. (2017), who explored how the participants would respond to the consistency of choice between themselves and the other player. In their study, consistent choices increase the participants’ motivational significance over the outcomes regardless of who make the choice first, as reflected in a more pronounced d-FRN. Taken together, the relationship between decision consistency and outcome evaluation has not reached a consistent conclusion, which might depend on the specific social context involved.

To sum up, decision consistency with others is a piece of important social information. Previous studies have begun to uncover the evaluation process of such social information itself (Klucharev et al., 2009; Nook and Zaki, 2015; Wang et al., 2019; Zheng et al., 2021) as well as its influence on the following outcome evaluation (Gehring and Willoughby, 2002; Yeung and Sanfey, 2004; Bellebaum et al., 2010; Foti et al., 2011; Liao et al., 2011; San Martín, 2012). Our study aims to extend this line of studies by examining the effect of decision consistency in a social context where individual decisions are compared to those of the counterpart rather than a whole group. In addition to probing its outcome evaluation consequence, we explore whether this decision consistency would influence one’s anticipation level for the outcome, which was seldomly investigated in previous studies. Anticipation for the outcome is an important cognitive process in one’s decision-making, which can be tracked by the stimulus-preceding negativity (SPN). The SPN is a typical ERP component generally observed when one is waiting for the outcome of his/her choice to be revealed (Damen and Brunia, 1987; Foti and Hajcak, 2012), whose magnitude increases steadily as the outcome approaches (Brunia et al., 2012; Foti and Hajcak, 2012; Wang et al., 2017). The SPN is widely adopted to measure one’s motivational significance toward the upcoming outcome (Damen and Brunia, 1987; Masaki et al., 2006; Brunia et al., 2011; Murphy, 2016). For instance, Meng et al. (2016) explored how the task challenge level affects participants’ anticipation for performance results. It was found that in the optimal challenge condition, the participants have stronger intrinsic motivation to win and exhibit a higher expectation for the performance feedback, resulting in a larger SPN amplitude.

To achieve our research aims, we designed a two-player gambling task, in which two participants faced the same binary choice and were asked to make the decision in turn. The first mover made the binary choice without any prior social information, while in his/her view the choice he/she had made may serve as *a priori* information for the second mover to make a decision. We explored the first movers’ evaluation of social information and decision outcomes by recording their electroencephalogram (EEG) data. We mainly focused on three stages, including evaluation of the other’s choice (i.e., social information), feedback anticipation and feedback evaluation. In line with prior studies, we analyzed magnitudes of the FRN, SPN and d-FRN to learn about one’s cognitive processing in these stages, respectively. We predicted that when the first movers learned that their choices were inconsistent with their counterparts’, the underlying cognitive processes would be similar to those involved when they were inconsistent with the whole group. To be specific, an enlarged FRN would be observed when the first mover noticed that the second mover chose a different option. As for the SPN in the feedback anticipation stage, we predicted that the first mover would pay more sustained anticipatory attention to feedback when previous choices were inconsistent, which would produce a more pronounced SPN. Finally, we predicted that when the first mover noticed that the second mover followed his/her choice, he/she would perceive a higher sense of responsibility for the outcome, giving rise to a significantly more pronounced d-FRN in the outcome evaluation stage.

## Materials and Methods

### Participants

A total of 25 students (18 males, M_age_ = 22.84, SD_age_ = 2.54) from Zhejiang University participated in our experiment. All of them reported right-handedness with corrected to normal vision. They signed informed consent before the experiment and were properly paid for their participation. EEG of three participants were not fully recorded due to equipment malfunction. Another three participants’ EEG data were excluded because of too many artifacts. Finally, data of 19 participants were included in the analysis. This study was approved by the Ethics Commitment of Neuromanagement Laboratory at Zhejiang University.

### Materials and Procedure

There were two players in our experiment, namely the first mover and the second mover. In each trial, the players were first told to choose between two red packets presented on the left and right side of the screen. One of the red packets contained ￥50, and the other one contained ￥0. The first mover had the priority to choose one of the two red packets first. It is worth pointing out that while the first mover was granted the authority to make a choice ahead of the counterpart, the second mover could freely select either of the two red packets, even if it had been chosen by the first mover. In our study, two participants took part in the experiment at the same time and were told that their roles in the task were randomly determined by drawing lots according to the cover story. In fact, one of the participants was played by an experiment assistant, while the real participants whose EEG were recorded always played the role of first mover.

**FIGURE 1:**
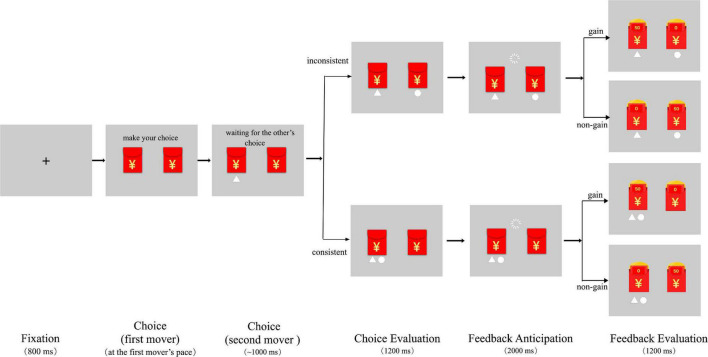
Timeline of a typical trial in the gambling task.

The experiment consisted of 3 blocks with a total of 140 trials. Each trial was divided into four stages, including choice, choice evaluation, feedback anticipation, and feedback evaluation (see Figure 1). The choice stage started with a fixation (800 ms) at the center of the screen, and then two red packets appeared on the left and right sides of the screen. The first mover (i.e., the real participant) was instructed to choose one of the two red packets at his/her pace by pressing 1 or 3 on the keyboard correspondingly. After the participant pressed a button, the chosen option would be highlighted by showing a triangle under the selected red packet. Then, the participant waited for the second mover to make a decision. The mean waiting time was programed to be 1,000 ms. Once the second mover finished selection, choices of both players were presented for 1,200 ms (i.e., the choice evaluation stage). Depending on whether the choices were consistent with each other, the trial fell into either the consistent or inconsistent choice condition. Then it came to the feedback anticipation stage, at which the participant should wait for 2,000 ms before the feedback outcome was revealed. Finally, both red packets were opened and outcomes were shown to the participants for 1,200 ms. Four kinds of feedback outcomes for the choice combinations of the two players (consistent/gain, inconsistent/gain, consistent/non-gain, inconsistent/non-gain) were provided at this feedback evaluation stage. The number of trials in these conditions was kept the same and the trials were randomly presented to the participants. The experimental task was prepared by the E-Prime 2.0 software package (Psychology Software Tools, Pittsburgh, PA, United States).

All participants were comfortably seated in a dimly lit, sound-attenuated, and electrically shielded room. Before the formal experiment, participants were asked to read the instructions and to complete five exercise trials to familiarize with the task procedure. The participants were informed that both players would be paid ￥35 for their participation, and three trials would be randomly selected. Their extra payoff would be calculated on a 1/10 scale based on their own outcomes in these three trials.

### Electroencephalogram Data Acquisition

We used the Neuroscan Synamp2 Amplifier (Scan 4.5, Neurosoft Labs, Inc., Sterling, VA, United States) with a cap of 64 scalp sites to record the EEG data (bandpass 0.05–70 Hz, sampling rate at 500 Hz). The electrode on the cephalic region was set as ground, and the left mastoid was set as an online reference. Besides, we recorded the horizontal Electrooculogram (EOG) by placing the electrodes at the left and right orbital rim, and the vertical EOG by placing the electrodes above and below the left eye. In addition, during the whole experiment, electrode impedance was maintained below 5 kΩ.

### Electroencephalogram Data Analyses

We pre-processed raw EEG data offline with Scan 4.5 and EEGLAB (Delorme and Makeig, 2004). Firstly, we re-referenced the EEG data to the average of the left and the right mastoids, filtered them with a 30 Hz low-pass filter (24 dB/Octave), and corrected the ocular artifacts. In addition, we excluded the epochs containing an amplifier clipping, bursts of electromyography activity, or an extreme amplitude (which exceeds ± 80 μV).

We were interested in how the first mover would evaluate the social information (the second mover’s choice), and then anticipate and evaluate the feedback of his/her choice. At choice evaluation and feedback evaluation stages, we focused on the FRN. EEG were segmented into epochs of 1200 ms, which lasted from 200 ms before the choice (or feedback) onset to 1,000 ms after their onset, with the first 200 ms serving as the baseline. Additionally, we averaged the EEGs by consistency (consistent vs. inconsistent) for choice evaluation, and by consistency × feedback (consistent/gain, inconsistent/gain, consistent/non-gain, and inconsistent/non-gain) for feedback evaluation. At the feedback anticipation stage, we focused on the SPN. EEG were segmented into epochs of 2,200 ms, which lasted from 2,200 ms before the onset of feedback to its onset, with the first 200 ms serving as the baseline. Similarly, we averaged the EEGs by choice consistency (consistent vs. inconsistent) for feedback anticipation.

**FIGURE 2:**
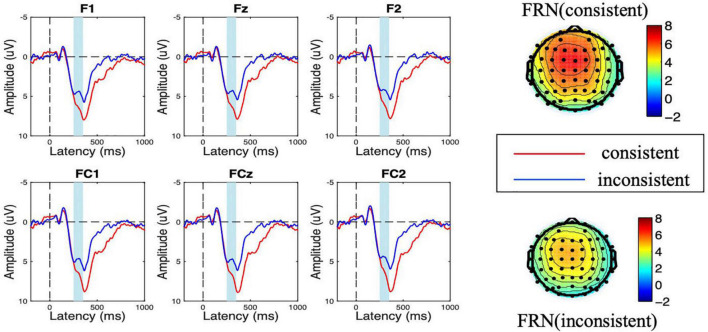
Amplitude of feedback-related negativity (FRN) at the choice evaluation stage for consistent vs. inconsistent conditions. The scalp topographic distribution of the FRN is provided.

**FIGURE 3:**
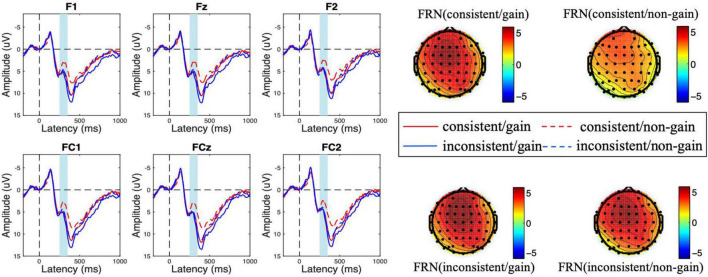
Amplitude of feedback-related negativity (FRN) at the feedback evaluation stage for consistency (consistent vs. inconsistent) and outcome valence (gain vs. non-gain) conditions. The scalp topographic distribution of the FRN is provided.

**FIGURE 4:**
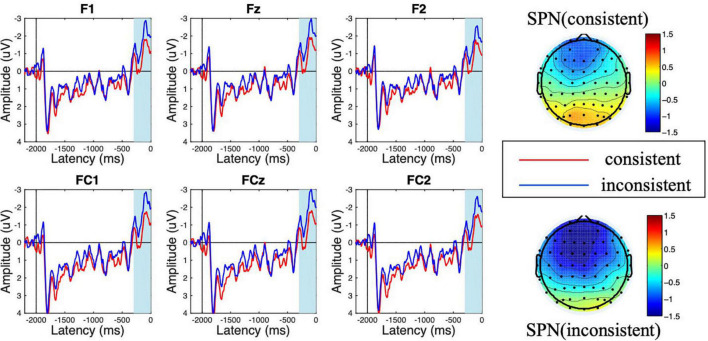
Amplitude of stimulus-preceding negativity (SPN) at the feedback anticipation stage for consistent vs. inconsistent conditions. The scalp topographic distribution of the SPN is provided.

As shown in Figures 2, 3, a frontal-distributed FRN-like component was observed both after choice and feedback presentations. The typical FRN appears at the frontal-central area and begins at about 200 ms after the outcome stimuli (Gehring and Willoughby, 2002; Zhou et al., 2010; Chen et al., 2012; Wang L. et al., 2016; Zheng et al., 2021). Therefore, the FRN during 250 ms to 350 ms at the fronto-central area (F1, Fz, F2, FC1, FCz, and FC2) was chosen for analyses of choice and feedback evaluations. Besides, as shown in the topographical map of Figure 4 and according to previous studies (Meng and Ma, 2015; Wang et al., 2018), we chose the SPN during −300 ms to feedback onset at the fronto-central region (F1, Fz, F2, FC1, FCz, and FC2) for the analysis of feedback anticipation.

Finally, the within-participant repeated measures ANOVA on the mean amplitudes of FRN (in the choice evaluation stage) and d-FRN with (consistency: consistent, inconsistent) × (electrodes: F1, Fz, F2, FC1, FCz, and FC2), FRN with (consistency: consistent, inconsistent) × (feedback valence: gain, non-gain) × (electrodes: F1, Fz, F2, FC1, FCz, and FC2), and SPN with (consistency: consistent, inconsistent) × (electrodes: F1, Fz, F2, FC1, FCz, and FC2) were conducted. Greenhouse–Geisser correction was applied when necessary.

## Results

### Feedback-Related Negativity at the Choice Evaluation Stage

During the choice evaluation stage, the ANOVA results showed that the main effect of consistency was significant [*F*_(1,18)_ = 11.782, *p* = 0.003, η^2^ = 0.396]. When the first movers noticed that the second movers chose the inconsistent option with them, a more negative FRN amplitude was elicited (M_inconsistent_ = 4.693 μV < M_consistent_ = 6.417 μV). Besides, the main effect of electrode was significant [*F*_(5,90)_ = 3.846, *p* = 0.026, η^2^ = 0.176], while the interaction effect of consistent × electrode was not significant [*F*_(5,90)_ = 0.120, *p* = 0.923, η^2^ = 0.007].

### Stimulus-Preceding Negativity at the Feedback Anticipation Stage

During the feedback anticipation stage, the ANOVA results showed that the main effect of consistency was significant [*F*_(1,18)_ = 18.232, *p* < 0.001, η^2^ = 0.503]. The SPN amplitude following inconsistent choices was more negative than that in the consistent choice condition (M_inconsistent_ = −1.619 μV < M_consistent_ = −0.816 μV). Besides, the main effect of electrode was significant [*F*_(5,90)_ = 3.840, *p* = 0.018, η^2^ = 0.176], while the interaction effect of consistency × electrode was not [*F*_(5,90)_ = 1.731, *p* = 0.170, η^2^ = 0.088].

### Feedback-Related Negativity at the Feedback Evaluation Stage

During the feedback evaluation stage, the ANOVA results showed that the main effect of consistency was marginally significant [*F*_(1,18)_ = 4.193, *p* = 0.055, η^2^ = 0.189] and the main effect of feedback valence was significant [*F*_(1,18)_ = 10.956, *p* = 0.004, η^2^ = 0.378]. The loss feedback elicited a more negative FRN amplitude (M_gain_ = 5.940 μV > M_loss_ = 4.607 μV). Besides, the interaction effect of consistency and feedback valence was significant [*F*_(1,18)_ = 4.475, *p* = 0.049, η^2^ = 0.199]. However, the main effect of electrode [*F*_(5,90)_ = 2.597, *p* = 0.094, η^2^ = 0.126], the interaction effect of consistency × electrode [*F*_(5,90)_ = 0.748, *p* = 0.494, η^2^ = 0.040], feedback × electrode [*F*_(5,90)_ = 1.716, *p* = 0.193, η^2^ = 0.087], and consistency × feedback × electrode [*F*_(5,90)_ = 0.746, *p* = 0.537, η^2^ = 0.040] were not significant.

We conducted a follow-up simple effect analysis and found that in the consistent condition, loss feedback elicited a more negative FRN amplitude [M_gain_ = 5.671 μV, M_loss_ = 3.552 μV, *F*_(1,18)_ = 20.875, *p* < 0.001, η^2^ = 0.537]. However, the effect of electrode [*F*_(5,90)_ = 1.766, *p* = 0.187, η^2^ = 0.089] and the interaction effect of valence × electrode [*F*_(5,90)_ = 5.590, *p* = 0.618, η^2^ = 0.032] were not significant. In the inconsistent condition, the valence effect did not reach significance [M_gain_ = 6.208 μV, M_loss_ = 5.662 μV, *F*_(1,18)_ = 0.772, *p* = 0.391, η^2^ = 0.041]. Besides, the effect of electrode [*F*_(5,90)_ = 3.152, *p* = 0.054, η^2^ = 0.149] and the interaction effect of valence × electrode [*F*_(5,90)_ = 2.334, *p* = 0.088, η^2^ = 0.115] were not significant.

To clearly show the difference in feedback evaluation between consistent and inconsistent conditions, we conducted a 2 (consistency: consistent, inconsistent) × 6 (electrode: F1, Fz, F2, FC1, FCz, and FC2) repeated measure ANOVA on the d-FRN (FRN upon non-gains minus FRN upon gains). The results showed that the main effect of consistency was significant [*F*_(1,18)_ = 4.475, *p* = 0.049, η^2^ = 0.199]. Specifically, the d-FRN was significantly more negative in the consistent condition (M_consistent_ = −2.119 μV < M_inconsistent_ = −0.546 μV). The main effect of electrode [*F*_(5,90)_ = 1.716, *p* = 0.193, η^2^ = 0.087] and the interaction effect of consistency × electrode [*F*_(5,90)_ = 0.746, *p* = 0.537, η^2^ = 0.040] were not significant.

## Discussion

In this study, we designed a dual-player gambling task in a social comparison context to probe how individuals would evaluate the social information of consistency in their decisions, and how this social information would affect the following anticipation and evaluation of the feedback of their choice. Compared with the situation in which the second mover made the same choice as the first mover, when the second mover made a different choice, the first mover exhibited a more pronounced FRN in choice evaluation, an enlarged SPN in feedback anticipation, and a less pronounced d-FRN in feedback evaluation. These results indicate that people indeed care about the other player’s decisions in a social context, and that their reactions to the outcome of their own decisions would be heavily influenced by such social information, even though their outcomes are fully independent of the others’ decisions.

At the choice evaluation stage, we found a larger FRN when the participants (the first mover) noticed that the second mover made the opposite choice, suggesting that they regarded this inconsistency in choice as a conflict. This result is in line with previous studies which reported that individuals perceive the consistent choice with others as a recognition and reward (Klucharev et al., 2009; Nook and Zaki, 2015; Wang et al., 2019). Previous studies indicated that the FRN may originate from the anterior cingulate cortex (Kiehl et al., 2000; Holroyd et al., 2004; San Martín, 2012), and distinguishes between positive and negative feedback (Holroyd and Coles, 2002; Cohen et al., 2007; Meng et al., 2021). Besides the valence of the feedback, positive and negative feedback can be defined in terms of the discrepancy between the feedback and one’s expectation. Accordingly, previous studies consistently reported that the greater extent that the feedback deviates from what one expects, the larger the conflict, and the more negative FRN amplitude is evoked (Kim et al., 2012; San Martín, 2012; Hauser et al., 2014; Qi et al., 2018; Wang et al., 2019). In previous electrophysiological studies involving social interactions, the FRN has been identified as a signal that characterizes the evaluation of social information. Specifically, when people observe that their own opinions or behaviors are different from those of others, this social information would be regarded as a negative outcome and then elicit a larger FRN (Cialdini and Goldstein, 2004; Klucharev et al., 2009; Shestakova et al., 2012; Kimura and Katayama, 2013; Wang et al., 2019; Zheng et al., 2021). Such inconsistency indicates a deviation from the social norm (Cialdini and Goldstein, 2004), and represents the loss of a potential social reward (Kim et al., 2012). To sum up, the FRN is a neural signature of prediction error, which mirrors the conflict detection of human behaviors (Gehring and Willoughby, 2002; Holroyd et al., 2002; Nieuwenhuis et al., 2004; San Martín, 2012). Our result is in line with these previous studies, revealing that the FRN could track the conflict in choice behaviors between first and second movers. While we predicted to observe the same pattern if the two players made their choices independently, we consider that the sequential decision-making design might have enhanced the conflict perception. In this study, the first mover made the choice independently, while the second mover made the choice after observing the first mover’s decision. When the second mover chose a different option with the first mover, it seemed that the second mover trusted their own beliefs more and did not rely on the prompt given by the first mover. In such a situation, the first mover might receive a more negative signal, resulting in a more significant FRN in the choice evaluation stage.

At the feedback anticipation stage, we found a more pronounced SPN when waiting for the upcoming outcome feedback after inconsistent choices had been made. According to previous studies, the SPN is an index that can reflect the extent to which people look forward to the upcoming feedback, and a larger SPN will be elicited with higher anticipation (Damen and Brunia, 1987; Masaki et al., 2006; Brunia et al., 2011; Murphy, 2016). Our result indicated that the first mover exhibited a higher level of anticipation for the following outcome when the second mover chose the opposite red packet. Previous studies have suggested this anticipation to be modulated by the motivational significance of the feedback. For instance, Wang et al. (2017) measured the SPN when the participants were waiting for their outcomes after completing the multiplication task or the addition task. The researchers found that a larger SPN was evoked after completing the multiplication task compared with the addition task, and attributed this enhanced anticipatory state to the increased motivational significance of feedback after exerting greater effort. The current study set up a gambling task in a social context, where a social comparison should naturally occur. Since nobody would like to behave worse than others, the inconsistent choice would strengthen the perceived motivational significance of the feedback (Wang et al., 2018), and thus increase the anticipatory attention to the following outcome in our study.

Our SPN result in feedback anticipation could also be explained by the uncertainty level. Prior studies have found that uncertainty would modulate subjective anticipation toward the subsequent feedback (Foti and Hajcak, 2012; Seidel et al., 2015; Novak et al., 2016), and an enhanced SPN is generally observed when participants are more uncertain about their task performance (Catena et al., 2012; Meng et al., 2016; Wang et al., 2018). For example, in a previous study the researchers found that when the participants were more confident in completing the task, they felt lower uncertainty. Thus their anticipation of the result would be weakened, which was reflected in a smaller SPN (Novak et al., 2016). In another study, two players tried to resolve several knowledge quizzes in sequence (Wang et al., 2018). The authors manipulated the participants’ uncertainty by adjusting the difficulty of the questions. The results showed that in case of high uncertainty, the first mover was more looking forward to the other’s answer, resulting in a larger SPN. In addition, when the second mover’s choice was different from that of the first mover, a larger SPN was observed when the first mover waited for the outcome. This is because in the inconsistent condition, the uncertainty of the first mover would be further amplified and the eagerness of giving correct answers becomes stronger. Our study replicated the findings of these studies to a large extent. All in all, the motivational significance and uncertainty would both be increased when the first mover saw the second mover making the opposite choice, and then higher anticipation for the upcoming feedback was induced as manifested in an enlarged SPN.

At the feedback evaluation stage, our results demonstrated a larger FRN for negative (non-reward) feedback than positive (reward) feedback, which is consistent with a vast amount of previous studies revealing the feedback valence effect on the FRN (Gehring and Willoughby, 2002; Yeung and Sanfey, 2004; Bellebaum et al., 2010; Foti et al., 2011; Liao et al., 2011; San Martín, 2012). More relevant to the scope of this study, some studies indicated social factors to dramatically modulate outcome evaluation (Leng and Zhou, 2010; Kimura and Katayama, 2016). In line with these studies, our study also found that social information, i.e., the second mover’s choice, would affect the first mover’s outcome evaluation. To be specific, in the inconsistent condition we did not find a significant difference in FRN toward gains and non-gains. However, the consistent choice between the two players gave rise to a more pronounced FRN discrepancy (d-FRN). According to the motivational significance theory of FRN, the amplitude of d-FRN reflects one’s subjective evaluation of feedback. The more people care about the feedback, the larger d-FRN would be elicited (Gehring and Willoughby, 2002; Ma et al., 2011; Meng and Ma, 2015). For example, when the outcome of a decision is more motivationally relevant to the decision-maker, a larger d-FRN would be observed (Li et al., 2010; Ma et al., 2011; Yu and Sun, 2013; Koban and Pourtois, 2014; Kimura and Katayama, 2016; Fu et al., 2017). In our study, the second mover made the decision after the first mover and could see the choice made by the first mover. Thus, the first mover might believe that the second mover’s decision was influenced by his/her own. Once a consistent choice had been made, the first mover might feel responsible for the second mover’s outcome, and then integrate their outcomes together in outcome evaluation, which increased the eagerness and significance to receive a positive outcome (Fu et al., 2017). In other words, to the first movers, observing a consistent choice means that the second mover might have followed their choice. Thus, the first movers cared more about the overall outcome of the two players and regarded it as highly motivationally significant, resulting in a larger d-FRN. This explanation gets supported by several previous studies. For example, a study found that in the lower responsibility condition, the motivational significance of feedback results was weakened, resulting in a smaller d-FRN (Li et al., 2010). Besides, Kimura and Katayama (2016) suggested that in the non-cooperative situation, participants had higher responsibility for their own payoff, and thus exhibited a significantly larger d-FRN when evaluating the feedback. In conclusion, the perceived responsibility of the first mover in the consistent choice condition resulted in higher concern about the outcome feedback and thus elicited a more pronounced d-FRN.

Our study extends the existing literature on social influence and outcome evaluation by deepening our understanding in terms of how choice inconsistency would moderate the three stages of decision making. Specifically, our study reveals the cognitive process of participants’ anticipation for the outcome, which provides further evidence for the insight that inconsistency would enlarge uncertainty and anticipation for the positive outcome and is a major contribution of this study. In addition, our results indicate that even the social information provided by a single person could yield a similar effect with that of a group, which helps enrich the theory of social influence. Practically, our findings suggest that one’s follow-up decision-making experience could be modulated by the revelation of the other player’s choice, which is illuminating for the design of the decision procedure. However, it is worth noting that in this study we neglected to collect the subjective reported data of the participants’ motivation level, which could help support our hypotheses and enhance our conclusions. Besides, our study only examined the social influence of a single person. Future researchers can extend this study and directly compare the influence of one person with that of a group.

## Conclusion

In summary, in this study a dual-player gambling task was designed to examine how first movers would evaluate the social information regarding decision inconsistency, and how this inconsistency would influence their anticipation for and evaluation of the monetary outcome associated with the decision. The results revealed that inconsistency with the counterpart made the first mover perceive a conflict (as reflected in a more negative FRN), and had higher anticipation to learn about the outcome (as reflected in a more significant SPN). Besides, we found a larger d-FRN upon feedback in the consistent condition, indicating increased motivational significance during outcome evaluation, which might be the result of the participants’ higher responsibility perception for the outcome. Taken together, these results verify that the social information inferred by a single person’s behavior could exert similar influence on one’s cognitive processing with the social information inferred by a whole group’s behaviors. Besides, in addition to social information and outcome evaluation stages, our results show that the anticipation of outcomes is also heavily affected by the consistency in social information.

## Data Availability Statement

The data that support the findings of this study are available from the corresponding author upon reasonable request.

## Ethics Statement

The studies involving human participants were reviewed and approved by the Ethics Commitment of Neuromanagement Laboratory at Zhejiang University. The participants provided their written informed consent to participate in this study.

## Author Contributions

JZ: research conception, experiment design, data collection, data analysis, and writing the initial and final draft. LW: research project supervision, writing, and reviewing the final draft. LM: research conception, experimental design, data analysis, and writing the initial and final manuscript. All authors contributed to the article and approved the submitted version.

## Conflict of Interest

The authors declare that the research was conducted in the absence of any commercial or financial relationships that could be construed as a potential conflict of interest.

## Publisher’s Note

All claims expressed in this article are solely those of the authors and do not necessarily represent those of their affiliated organizations, or those of the publisher, the editors and the reviewers. Any product that may be evaluated in this article, or claim that may be made by its manufacturer, is not guaranteed or endorsed by the publisher.
